# Evaluating the Concordance Between ChatGPT and Multidisciplinary Teams in Breast Cancer Treatment Planning: A Study from Bosnia and Herzegovina

**DOI:** 10.3390/jcm14186460

**Published:** 2025-09-13

**Authors:** Sefika Umihanic, Hedim Osmanovic, Nejra Selak, Dijana Kopric, Asija Huseinbasic, Erna Sehic-Kozica, Belma Babic, Fadil Umihanic

**Affiliations:** 1 Department of Oncology and Radiotherapy, University Clinical Center Tuzla, 75000 Tuzla, Bosnia and Herzegovina; umihanics@gmail.com (S.U.); dijana.kopric@ukctuzla.ba (D.K.); asija.huseinbasic@gmail.com (A.H.); belma996@gmail.com (B.B.); 2 Faculty of Natural Sciences and Mathematics, University of Tuzla, 75000 Tuzla, Bosnia and Herzegovina; hedim.osmanovic@untz.ba; 3 Department of Pathology, University Clinical Center Tuzla, 75000 Tuzla, Bosnia and Herzegovina; 4 Department of Oncology and Radiotherapy, Cantonal Hospital Zenica, 72000 Zenica, Bosnia and Herzegovina; e.sehickozica@gmail.com; 5 Cerrahpaşa Faculty of Medicine, Istanbul University-Cerrahpaşa, 34390 Istanbul, Turkey; fadil.umihanic@ogr.iuc.edu.tr

**Keywords:** artificial intelligence, ChatGPT, breast cancer, oncology, multidisciplinary team, low- and middle-income countries, clinical decision support

## Abstract

**Background/Objectives**: In many low- and middle-income countries (LMICs), including Bosnia and Herzegovina, oncology services are constrained by a limited number of specialists and uneven access to evidence-based care. Artificial intelligence (AI), particularly large language models (LLMs) such as ChatGPT, may provide clinical decision support to help standardize treatment and assist clinicians where oncology expertise is scarce. This study aimed to evaluate the concordance, safety, and clinical appropriateness of ChatGPT-generated treatment recommendations compared to decisions made by a multidisciplinary team (MDT) in the management of newly diagnosed breast cancer patients. **Methods**: This retrospective study included 91 patients with newly diagnosed, treatment-naïve breast cancer, presented to an MDT in Bosnia and Herzegovina in 2023. Patient data were entered into ChatGPT-4.0 to generate treatment recommendations. Four board-certified oncologists, two internal and two external, evaluated ChatGPT’s suggestions against MDT decisions using a 4-point Likert scale. Agreement was analyzed using descriptive statistics, Cronbach’s alpha, and Fleiss’ kappa. **Results**: The mean agreement score between ChatGPT and MDT decisions was 3.31 (SD = 0.10), with high consistency across oncologist ratings (Cronbach’s alpha = 0.86). Fleiss’ kappa indicated moderate inter-rater reliability (κ = 0.31, *p* < 0.001). Higher agreement was observed in patients with hormone receptor-negative tumors and those treated with standard chemotherapy regimens. Lower agreement occurred in cases requiring individualized decisions, such as low-grade tumors or uncertain indications for surgery or endocrine therapy. **Conclusions**: ChatGPT showed high concordance with MDT treatment plans, especially in standardized clinical scenarios. In resource-limited settings, AI tools may support oncology decision-making and help bridge gaps in clinical expertise. However, careful validation and expert oversight remain essential for safe and effective use in practice.

## 1. Introduction

Breast cancer is the most common malignancy among women and the second leading cause of cancer-related death, following lung cancer [1]. Women aged 45 to 65 years, a demographic undergoing hormonal changes associated with the peri- and postmenopausal period, are the most vulnerable [2]. Although the overall five-year survival rate for breast cancer is 89.2%, the stage at diagnosis has a major impact, ranging from 98% in stage I to only 24% in stage IV, highlighting the urgent need for early detection and effective treatment [3].

Delays in cancer diagnosis and treatment are major contributors to high mortality in low- and middle-income countries (LMICs), largely due to critical shortages of specialized professionals, including pathologists and oncologists, as well as limited diagnostic infrastructure [4]. Pathologist-to-population ratios in many LMICs are significantly lower than in high-income countries, leading to delays or inaccuracies in diagnosis. Access to radiotherapy remains limited, with severe shortages in equipment and trained personnel, while essential cancer medications are often unaffordable or unavailable. In 2019, Bosnia and Herzegovina had only 15 oncologists per 10,000 cancer patients, ranking in the mid-range among Eastern European countries, underscoring workforce limitations in the region [5]. More recent professional estimates suggest that the total number of oncologist may have been around 29 by the end of 2024, although no national registry currently exists to provide official confirmation (private correspondent), which remains low. Addressing these systemic barriers is crucial to improving timely, equitable, and effective cancer care.

In light of these challenges, there is growing interest in scalable, technology-based solutions that can help in clinical decision-making and help alleviate pressure on overburdened healthcare systems. Artificial intelligence (AI), particularly large language models (LLMs), is increasingly being explored for its potential to support cancer care through advanced data processing, contextual understanding, and decision-making assistance. LLMs like ChatGPT, Claude2, and BioMedLM have demonstrated promising capabilities across various tasks [6]. These models were evaluated in diverse scenarios, including breast cancer management, precision oncology for genetically altered tumors, and patient-reported symptom monitoring, revealing both their strengths and limitations. Similarly, in a simulated precision oncology setting, LLMs were able to generate unique, sometimes useful treatment options, although their outputs often lacked sufficient supporting evidence and were easily distinguishable from expert recommendations. Additionally, tools like ChatGPT-4 have shown promise in analyzing electronic patient-reported outcomes, offering practical suggestions to improve care and communication. When reviewed by clinicians, these AI-assisted insights were generally seen as supportive, particularly in addressing diet, symptoms, and emotional well-being [7]. These findings suggest that LLMs can serve as valuable adjunct tools in oncology, especially in well-defined clinical pathways, provided their outputs are carefully validated and contextualized by experts.

Especially regarding the limitations of LMICs, AI-based platforms offer promising solutions by providing accessible, evidence-based treatment recommendations and supporting clinicians, especially where oncology expertise is scarce [8]. These tools can help standardize care, reduce cognitive burdens on less experienced physicians, and promote knowledge sharing across regions.

This retrospective study evaluates treatment recommendations generated by ChatGPT-4.0 and compares them with multidisciplinary team (MDT) decisions for newly diagnosed breast cancer patients in Bosnia and Herzegovina. Four board-certified oncologists, including both MDT members and external reviewers, independently assessed the concordance of ChatGPT’s suggestions using a structured rating approach. The primary aim was to quantify the agreement between AI-generated outputs and expert-led MDT treatment plans in a real-world low- and middle-income country (LMIC) setting. 

## 2. Materials and Methods

This retrospective study was conducted at the Clinic for Oncology and Radiotherapy, University Clinical Center Tuzla, and approved by the institutional Ethics Committee (Approval No. 02-09/2-116-3/25). The study included patients with newly diagnosed breast cancer, presented for the first time to the MDT specialized in breast cancer care, between 1 January and 31 December 2023.

Inclusion criteria were patients with a new diagnosis of breast cancer who had not received any prior treatment and were presented to the MDT for initial management planning. Exclusion criteria included (1) incomplete clinical data; (2) multiple synchronous primary cancers; (3) local recurrence; (4) age younger than 18 or older than 89 years; and (5) participation in any clinical trial.

Demographic and clinicopathological variables were extracted from patient records, including sex, age, menopausal status, estrogen receptor (ER) and progesterone receptor (PR) status, HER2 status, tumor grade (in situ, G1 G2 and G3), and Ki-67 proliferation index.

For each case, treatment recommendations from the MDT were documented. Potential modalities included chemotherapy, hormone therapy, radiotherapy, anti-HER2 therapy, surgery, or multimodal approaches. ChatGPT (version 4.0, OpenAI, San Francisco, CA, USA) was independently queried with the corresponding patient data to generate its treatment recommendations.

An example of the ChatGPT prompt is as follows:

“A 45-year-old female with invasive ductal breast carcinoma, cT2b cN0, 80% estrogen receptor expression, 70% progesterone receptor expression, HER2 3+, Ki-67 of 25%, and grade 2 differentiation, without comorbidities. Proposed treatment?”

To prevent bias from prior responses, each ChatGPT query was submitted in a fresh chat session after clearing the previous chat history.

Four board-certified oncologists from three reference centers independently evaluated the treatment plans generated by both the MDT and ChatGPT. Two oncologists were members of the MDT, while the other two were from an external institution to reduce institutional bias.

Each ChatGPT-generated recommendation was analyzed in comparison with the MDT proposal. We correlated each individual diagnostic parameter with both ChatGPT and MDT recommendations to ensure that all clinically relevant data were considered in treatment suggestions. Subsequently, we analyzed the overall agreement between the ChatGPT and MDT treatment proposals. Inter-oncologist agreement was also assessed.

Each expert assessed ChatGPT’s recommendation in comparison with the MDT’s plan using a 4-point Likert scale (1: Strongly disagree; 2: Disagree; 3: Agree; 4: Strongly agree).

Each oncologist’s ratings were scored individually, and total scores were calculated to reflect agreement levels. Agreement between ChatGPT-generated treatment recommendations and MDT decisions was evaluated using descriptive statistics, Cronbach’s alpha, and Fleiss’ kappa coefficient. All statistical analyses were performed using R software (version 4.5.1; R Foundation for Statistical Computing, Austria). A *p* value < 0.05 was considered statistically significant.

## 3. Results

A total of 91 newly diagnosed breast cancer patients were included, with a median age of 60 years (IQR: 50–70). All patients were female. The majority were postmenopausal (84%) and had invasive ductal carcinoma (83.5%). Most tumors were hormone receptor-positive (ER+: 76.9%, PR+: 69.2%) and HER2-negative (85.7%), while 14.3% were HER2-positive. 40.7% patients had no comorbidity. Among those with comorbidities, the most common was arterial hypertension (*n* = 43), followed by diabetes mellitus (*n* = 14) and hyperlipidemia or hypothyroidism (both *n* = 5). The least common comorbidities included valvular heart disease, history of venous thromboembolism, peripheral neuropathy, and hepatitis (each *n* = 1, 1%). Clinical and demographic data are given in Table 1.

**Figure 1:**
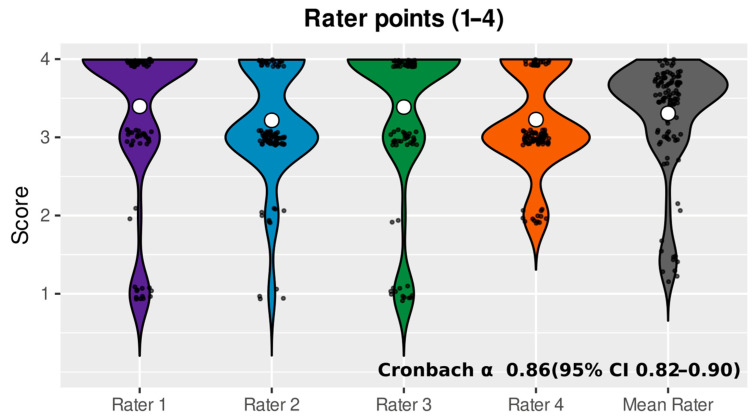
Violin plot of rater scores. Violin plots display the distribution of agreement scores for each of the four raters and their average. Scores ranged from 1 (strongly disagree) to 4 (strongly agree). White dots indicate the mean score for each rater. Most scores clustered around 3 and 4, suggesting a general trend toward high agreement with the ChatGPT recommendations. The high internal consistency among raters is confirmed by a Cronbach’s alpha of 0.86 (95% CI: 0.82–0.90), indicating very good inter-rater reliability.

The overall agreement between ChatGPT-generated treatment suggestions and the multidisciplinary team (MDT) recommendations was high, with a mean rating score of 3.31 (SD = 0.10) across all patients (Figure 1). Scores were consistent across raters, with mean values ranging from 3.22 to 3.40. Ratings below the agreement threshold (score < 2) were observed in roughly 11–13% of cases per rater. Inter-rater reliability was strong, with a Cronbach’s alpha of 0.86, while Fleiss’ kappa indicated moderate agreement (κ = 0.31, *p* < 0.001).

Agreement varied by clinical subgroup. Higher agreement was observed in patients with ER-/PR- tumors and those receiving standardized neoadjuvant chemotherapy regimens, such as TCHP or AC-T + Carboplatin. Conversely, lower agreement was noted in subgroups requiring more individualized judgment, including grade 1 tumors and cases with uncertain indications for surgery or endocrine therapy. Detailed subgroup results are summarized in Table 2, which provides mean scores, variability, and proportion of lower vs. higher ratings across relevant clinical and treatment-related categories.

## 4. Discussion

In this study of 91 newly diagnosed breast cancer patients, we observed a high overall concordance between AI-generated treatment recommendations and those of a multidisciplinary oncology team. Agreement scores were consistent across four oncologist raters, ranging from a mean of 3.22 to 3.40, with inter-rater reliability indicated by a high Cronbach’s alpha of 0.86 and moderate Fleiss’ kappa of 0.31 (*p* < 0.001). Notably, concordance was greater in clinical scenarios involving receptor-negative tumors and neoadjuvant chemotherapy regimens, whereas more individualized cases, such as low-grade tumors or uncertain surgical and endocrine treatment decisions, showed lower alignment.

Several groups have recently evaluated ChatGPT in oncology. One of the first studies on this topic was by Sorin et al., who tested ChatGPT-3.5 in 10 breast cancer patients [9]. Two radiologists evaluated its output compared with MTB decisions for recommendations, explanations, and summaries, with 70% of ChatGPT’s suggestions similar to the board’s decisions. Mean scores on a 1–5 scale ranged from 3.7 to 4.6, indicating moderate to high agreement. Still, they noted missing key information in one case and the absence of radiologist input in the decision process.

Griewing et al. analyzed 20 generic breast cancer scenarios, also using ChatGPT-3.5. [10] The highest concordance was for chemotherapy, and the lowest for genetic testing. The authors highlighted that ChatGPT-3.5, trained only until September 2021, could not reflect current guidelines. In fact, the model sometimes gave misleading advice, for example, recommending BRCA testing based on a sister-in-law’s cancer history or omitting re-excision for DCIS with a 0.01 mm margin. Neither this nor any previous study provided the model with updated guidelines.

More recently, Deng et al. evaluated ChatGPT-4 in five simulated sarcoma cases [11]. Using imported guidelines and optimized prompting, they reported a mean score of 3.76/5 (75.2% of the maximum), again reflecting only moderate concordance. Even with a newer model and a different prompting method, the study concluded that ChatGPT is not yet fully reliable for independent clinical recommendations. In our study, which used a 4-point Likert scale, the mean agreement was 3.31, also consistent with moderate concordance. Comparable results were reported in another study evaluating ChatGPT’s treatment suggestions, where an overall concordance rate of 83% was observed across 93 cancer cases [12]. Agreement was highest for standardized treatment decisions such as neoadjuvant, surgical, and adjuvant therapy, while lower concordance was seen in HCC cases and areas like systemic therapy, follow-up, and loco-regional interventions, reflecting weaker alignment in less standardized scenarios. Although the scales used (1–5, binary, and 1–4 in our work), the GPT version, and the patient subsets differ, the overall pattern is consistent and confirmed by our study: ChatGPT performs well in guideline-driven, standardized cases but is less reliable when individualized clinical judgment is required.

Other studies have also evaluated AI in oncology beyond GPT. A retrospective analysis comparing Watson for Oncology (WFO) with a multidisciplinary tumor board in gastric cancer reported an overall concordance of 86.9%, with the highest agreement in early-stage disease and the lowest in stage IV [13]. Concordance was influenced by age, performance status, and disease stage. Discrepancies were linked to local guideline differences (stages I and IV) and patient-related factors such as age over 80 and poor performance status (stages II and III). In our breast cancer cohort, agreement with ChatGPT was similarly high, especially in receptor-negative tumors and standard chemotherapy settings. Lower concordance was observed in more clinically complex or uncertain scenarios, suggesting that AI aligns best with clear, guideline-driven treatment pathways but may struggle in more individualized cases. In a breast cancer study in China [14], Watson for Oncology’s treatment recommendations matched well with clinical decisions for postoperative therapy but had only 27.5% concordance for metastatic chemotherapy. This was partly due to recommending CDK4/6 inhibitors unavailable in China and preferring single-agent chemotherapy, while 43% of patients received combination therapy, although the NCCN guidelines does not point to evidence that combination regimens are superior to single agents.

In oncology, AI is already making inroads, particularly in breast cancer, through computer-aided detection in mammography screening [15] and in radiation oncology [16]. The use of AI in healthcare broadly refers to algorithms that simulate human cognitive functions to analyze complex clinical data. Similar AI applications have shown promise in treatment decision-making for cancers in other anatomical sites, such as the esophagus and lungs, including decisions on surgery, radiotherapy, and systemic therapy [17,18]

Although ChatGPT shows promise as a decision support tool, several limitations must be considered when integrating it into clinical practice. First, its outputs are shaped by the data it was trained on, which may carry inherent biases, such as underrepresentation of certain patient populations or diseases, potentially leading to skewed or less accurate recommendations [19]. Secondly, ChatGPT often lacks the ability to fully grasp clinical context, and while its suggestions may appear logical, they may miss critical nuance or depth needed for sound medical judgment. Therefore, human oversight remains essential. Clinicians must critically evaluate AI-generated outputs to ensure they are safe, appropriate, and tailored to each individual patient’s situation.

As the integration of AI into healthcare accelerates, ethical and regulatory considerations have become increasingly important to ensure its responsible use. The WHO highlights the need for safe, ethical, and effective AI in healthcare, urging collaboration among developers, regulators, clinicians, and patients, with a focus on high-quality data to prevent bias. As of 1 August 2024, the EU’s AI Act enforces the first comprehensive regulation to ensure transparency and oversight for high-risk AI systems in healthcare [20].

Strengths of this study include its real-world design in a LMIC setting, evaluating ChatGPT’s performance in actual multidisciplinary decision-making workflows. The inclusion of patients with newly diagnosed, treatment-naïve breast cancer enhances clinical relevance. By involving board-certified oncologists from three reference centers, including both MDT members and external reviewers, the study minimized institutional bias and allowed for robust inter-rater agreement analysis. The methodology also reflects practical application scenarios for AI-assisted decision support in resource-constrained environments.

Limitations include the single-country setting, which may limit generalizability to other healthcare systems, especially in LMICs. The study relied on structured clinical data; however, additional contextual details (e.g., patient preferences, psychosocial factors, etc.) were not included in the ChatGPT prompts and may influence treatment planning in real clinical scenarios. While ChatGPT recommendations were generated using version 4.0, rapid model updates may affect reproducibility over time. Furthermore, only one AI model was evaluated. Other LLMs, such as Claude or Bard, may perform differently; nevertheless, we focused on ChatGPT due to its broad accessibility, widespread use, and free availability, making it particularly relevant in LMIC settings.

Our study provides the first empirical evidence of this pattern in a real-world LMIC setting. Importantly, we quantified the degree of concordance between ChatGPT and MDT decisions, demonstrating very high reliability in straightforward cases. These finding highlights AI’s potential role in supporting oncology care where specialist resources are limited, by ensuring consistency in routine decision-making. At the same time, our results underscore the need for continued human oversight in more complex or borderline situations, where individualized clinical judgment remains irreplaceable. Our study aims to familiarize clinicians with both the potential and limitations of AI as a support tool for MDTs in breast cancer treatment. Improving the explainability of AI models is essential for fostering trust among clinicians and encouraging broader adoption.

## 5. Conclusions

This study demonstrates that large language models like ChatGPT can provide treatment recommendations for newly diagnosed breast cancer patients that are largely concordant with multidisciplinary team decisions, particularly in standardized, chemotherapy-oriented regimens. Conducted in a real-world low- and middle-income countriessetting, our findings highlight the potential role of AI as an adjunct decision-support tool in oncology, especially where specialist resources may be limited. While not a replacement for clinical expertise, ChatGPT may offer value in streamlining care, supporting less experienced providers, and enhancing access to evidence-based guidance.

However, its current limitations in accuracy and clinical judgment are significant. Further research and development are essential to optimize AI tools for reliable use in medical decision-making.

## Figures and Tables

**Table 1:** Clinical and demographic data.

Variables		N (%)
Age (median (IQR)) (years)		60 (50–70)
Marital status	Married	71 (78.0)
	Widowed	19 (20.9)
	Divorced	1 (1.1)
Menopausal status	Premenopausal	6 (6.6)
	Postmenopausal	76 (83.5)
	Unknown	9 (9.9)
Partus	Yes	42 (46.2)
	Nno	18 (19.8)
	Unknown	31 (34.0)
Comorbidities	0	37 (40.7)
	1	30 (33.0)
	2	13 (14.3)
	3+	11 (12.0)
Tumor type	Ductal	66 (72.5)
	Lobular	11 (12.1)
	Other	14 (15.4)
Histological grading (NG)	in situ	1 (1.1)
	1	3 (3.3)
	2	61 (67.0)
	3	23 (25.3)
	Unknown	3 (3.3)
ER receptor	Positive	70 (76.9)
	Negative	21 (23.1)
PR receptor	Positive	63 (69.2)
	Negative	28 (30.8)
Her2 receptor	Positive	13 (14.3)
	Negative	78 (85.7)
Ki67	≤20	52 (57.1)
	>20	39 (42.9)

**Table 2:** Subgroup analysis of agreement between ChatGPT-generated and MDT treatment recommendations. Results are presented as mean Likert scores (1–4), standard deviation (SD), and proportion of lower (≤2) vs. higher (>2) ratings across clinically relevant variables.

Category	Group	Number of Patients	Mean Score	SD	Rate ≤ 2 (%)	Rate > 2 (%)
**Partus**	Yes	42	3.45	0.18	3.0	97.0
	No	18	3.49	0.27	4.2	95.8
	Unknown	31	2.94	0.12	32.3	67.7
**Tumor type**	Ductal	66	3.22	0.06	15.5	84.5
	Lobular	11	3.52	0.18	4.5	95.5
	Other	14	3.38	0.20	8.9	91.1
**Histological grading**	In situ	1	2.75	0.33	25	75
	1	3	2.84	0.12	33.3	66.7
	2	61	3.26	0.09	12.7	87.3
	3	23	3.45	0.15	8.7	91.3
**Histological grading**	Unknown	3	3.00	0.19	33.3	66.7
**ER receptor**	Positive	70	3.22	0.05	13.1	86.9
	Negative	21	3.52	0.18	2.5	97.5
**PR receptor**	Positive	63	3.14	0.04	16.7	83.3
	Negative	28	3.59	0.22	5.6	94.4
**HER2 receptor**	Positive	13	3.5	0.32	9.6	90.4
	Negative	78	3.24	0.05	13.8	86.2
**Ki-67**						
	≤20	52	3.29	0.07	12.5	87.5
	>20	39	3.29	0.12	14.1	85.9
**Neoadjuvant AC-T + carboplatin**	Yes	2	3.68	0.48	3.6	96.4
	No	55	3.22	0.13	15.9	84.1
	Indecisive	44	3.20	0.06	14.2	85.8
**Neoadjuvant AC-T + platinum**	Yes	2	3.12	0.48	12.5	87.5
	No	46	3.32	0.12	13.0	87.0
	Indecisive	43	3.25	0.04	12.2	87.8
**Surgery**	Yes	76	3.32	0.11	12.5	87.5
	No	7	3.36	0.14	7.1	92.9
	Indecisive	8	2.91	0.24	25	75
**Radiotherapy**	Yes	47	3.38	0.13	10.6	89.4
	No	6	3.58	0.29	0	100
	Indecisive	38	3.12	0.04	18.4	81.6
**Endocrine therapy**	Yes	63	3.36	0.09	7.5	92.5
	No	23	3.18	0.11	21.7	78.3
	Indecisive	5	2.75	0.25	45	55
**Palliative care**	Yes	6	3.42	0.29	4.2	95.8
	No	85	3.28	0.07	13.8	86.2
**FISH**	Yes	4	3.38	0.75	18.9	81.1
	No	87	3.28	0.07	12.9	87.1
**Neoadjuvant TCHP**	Yes	2	3.88	0.25	0	100
	No	50	3.27	0.12	15	85
	Indecisive	39	3.26	0.05	11.5	88.5

## Data Availability

Data are available from the corresponding author upon reasonable request.

## Abbreviations

The following abbreviations are used in this manuscript:

AI	Artificial Intelligence
LLM	Large Language Model
MDT	Multidisciplinary Team
LMIC	Low- and Middle-Income Country
ER	Estrogen Receptor
PR	Progesterone Receptor
HER2	Human Epidermal Growth Factor Receptor 2
AC-T	Doxorubicin (Adriamycin), Cyclophosphamide, followed by Taxane
TCHP	Docetaxel, Carboplatin, Trastuzumab, and Pertuzumab
FISH	Fluorescence In Situ Hybridization
